# Risk factors for ectopic pregnancy in a population of Cameroonian women: A case-control study

**DOI:** 10.1371/journal.pone.0207699

**Published:** 2018-12-12

**Authors:** Yvette Audrey Assouni Mindjah, Félix Essiben, Pascal Foumane, Julius Sama Dohbit, Emile Telesphore Mboudou

**Affiliations:** 1 Faculty of Medicine and Biomedical Sciences, University of Yaoundé I, Yaoundé, Cameroon; 2 Department of Obstetrics and Gynaecology, Faculty of Medicine and Biomedical Sciences, University of Yaoundé I, Yaoundé, Cameroon; Massachusetts General Hospital, UNITED STATES

## Abstract

**Objective:**

To identify the risk factors for ectopic pregnancy (EP) in a population of Cameroonian women.

**Sample and methods:**

We performed a matched case-control study; 88 women with diagnosed EP (cases), and 176 women with first trimester intrauterine pregnancy (IUP) (controls), who underwent questionnaires. Odds Ratio (OR) and 95% confidence intervals (CIs) were calculated and adjusted for potential confounding factors via multivariate logistic regression analysis.

**Results:**

Of the fifteen identified risk factors, 4 were independently associated with increased odds of EP: prior pelvic inflammatory disease (PID) (adjusted odds ratio [AOR] 13.18; 95% CI 6.19–27.42), followed by current use of levonorgestrel-only pills for emergency contraception (LNG-EC) (AOR 10.15; 95% CI 2.21–46.56), previous use of depot medroxyprogesterone acetate (DMPA) (AOR 3.01; 95% CI 1.04–8.69) and smoking at the time of conception (AOR 2.68; 95% CI 1.12–6.40).

**Conclusion:**

The present study confirms the wide variety of EP’s risk factors. Moreover, some new findings including current use of LNG-EC, previous use of DMPA, smoking at the time of conception are noteworthy. Thus, in our limited resources country where prevention remains the cornerstone for reducing EP chances of occurrence, clinicians should do enough counselling, especially to women with known risk factors. The necessity to facilitate access to more equipment to enable early diagnosis of EP is very crucial and should be seriously considered, in order to reduce the burden of EP in Cameroonian women.

## Introduction

Ectopic pregnancy (EP) is the main cause of maternal death during the first trimester of pregnancy and accounts for approximately 10% of all pregnancy-related deaths [1,2]. In African developing countries, where most of the women tend to present at the rupture stage (with an unstable hemodynamic state), it is an important cause of maternal deaths, with a case fatality rates around 1–3%; 10 times higher than reported in industrialized countries [3–5]. In Ghana, 8.7% of maternal deaths were due to EP [5]. Recently in Cameroon, it was reported to be responsible for 12.5% of maternal deaths [6]. This could be explained by late diagnosis, due to the unavailability of diagnostic means in our context, although the early diagnosis of EP became possible with various examinations notably transvaginal ultrasonography and quantitative measurement of the β subunit of human chorionic gonadotropin (β-hCG) [5,7]. A delay in diagnosis most often leads to severe complications (rupture and hemoperitoneum) and consequently to surgical treatment by laparotomy with salpingectomy [5,8–11].

Over the last few decades, the incidence of EP has steadily increased around the world. In the Western countries, it varies between 1–2% [9]. Yet this incidence is higher in developing countries, especially in Cameroon where it reaches 4.23% [7,12,13]. Some Sweden studies have demonstrated that the increased in EP’s incidence was strongly associated to a rise in the incidence of PID and some sexually transmitted infections (STIs) [14,15].

There are other risk factors that have been associated with EP including prior EP, previous tubal surgery, documented tubal pathology, history of infertility, cigarette smoking, assisted reproduction technologies (ARTs), multiple lifetime sexual partners, older maternal age, and in utero diethylstilbestrol (DES) exposure [16–20]. Some meta-analyses found that oral contraceptive pills (OCPs), intrauterine devices (IUDs), and female sterilization could increase the risk of EP to different degrees in cases of contraceptive failure [16,21]. The use of levonorgestrel-only pills for emergency contraception (LNG-EC) at the time of the conception has recently been identified as a risk factor for EP [19,20]. In Cameroon, where the era is for the promotion of family planning nationwide through the increased use of modern contraceptives, it would be important to evaluate their involvement and strength in EP’s occurrence in our context.

Further, given that the mortality and morbidity associated with EP are related to the length of time required for diagnosis, increased awareness and knowledge on its risk could help by providing better prediction and prevention in at risk-women. Moreover, this could enable an early and accurate diagnosis prior to the rupture, resulting in a reduction in the need for surgery and some complications.

## Materials and methods

This study was approved by the Institutional Ethical Review Board for Human Health, under the ethical clearance No 314/CIERSH/2016, and by the Institutional Ethical Review Board of the Faculty of Medicine and Biomedical Sciences, University of Yaoundé I, Yaoundé, Cameroon under the ethical clearance No 166/CIER/2017. Administrative authorizations were obtained in both hospitals involved prior to the beginning of the study. A written informed consent was obtained from each participant before recruitment.

### Participants and methods

This case-control study was carried out in the two main referral hospitals for gynaecologic and obstetric emergencies in the Yaoundé metropolis–Yaoundé Gynaeco-Obstetric and Pediatric Hospital (YGOPH), Principal Maternity of the Yaoundé Central Hospital (YCH)—from November 1st, 2016 to April 30, 2017. According to the American College of Obstetricians and Gynecologists Practice Bulletin [22], the diagnosis and location of pregnancy were confirmed at operation for EP patients who underwent surgical treatment. Transvaginal ultrasonography combined to the measurement of serum β-HCG levels was used to confirm EP’s diagnosis for patients who received medical treatment. During the study period, all women who had been diagnosed with EP in the inpatient department of gynaecology of each hospital were recruited in the case group (EP group). Women with a first trimester intrauterine pregnancy (IUP) at the prenatal clinic of these hospitals during this period, matched for age at a 1:2 ratio were included as controls (intrauterine pregnancy [IUP] group).

The investigator was responsible for data collection from the participants during an interview, by using a questionnaire. They were assured of confidentiality, as some of the questions were very private. All women who provided incomplete information were excluded. After excluding 6 participants, a total of 264 women were enrolled in the case-control study, including 88 cases (EP group) and 176 controls (IUP group); a response rate of 97.78%.

Information collected included sociodemographic characteristics (age, educational level, marital status, professional category, and smoking at the time of conception). The professional category was defined according to the Cameroonian Nomenclature of Trades, Jobs, and Professions [23]. There were questions included details about past relevant reproductive and gynaecologic histories (including previous miscarriage, prior induced abortion, parity, prior *Chlamydia Trachomatis* **(**CT**)** infection, prior PID, prior EP, previous infertility, documented tubal pathology, previous tubal surgery, age at sexual debut, lifetime’s number of sexual partners) and about surgical history. Previous and current use of contraceptives (including intrauterine devices [IUDs], combined oral contraceptives [COCs], progestin-only pills [POPs], LNG-EC, progestin-only implants and progestin-only injectable of depot medroxyprogesterone acetate (DMPA). According to Cheng Li et al. [24], a woman was defined as a previous user of a given contraceptive method if she had used the method in the previous cycle and as a current user if she had used the method in the current cycle.

### Statistical analysis

All statistical analyses were performed using IBM SPSS Statistics software, version 23. The frequency of distribution of each variable was examined according to the case and control groups. Crude odds ratios (ORs) of each variable with their 95% confidence intervals (CIs) were calculated in the bivariate analysis. When we explored the association between risk of EP and prior CT infection, documented tubal pathology there were missing values; these were eliminated from the analysis. All *p values* were calculated using statistical Chi-Square and Fisher’s exact tests when necessary. Values less than 0.05 were considered statistically significant. Variables significantly associated with EP by bivariate analysis were included as candidates in the multivariable logistic regression analysis to adjust for potential confounders and calculate the adjusted odds ratio (AOR) with the aim of identifying the independent risk factors for EP.

## Results

During the study period, a total of 90 women with EP were enrolled. One hundred and eighty women with IUP were recruited to be included in the control group. After women providing incomplete information were excluded, 264 women with first trimester pregnancies were included in the study; with a total of 88 final cases (EP group) and 176 controls (IUP group).

### Socio-demographic factors

Table 1 presents the sociodemographic characteristics between the two groups. EP women were more likely to have a high school educational level (p<0.001). Because of the matching criteria of cases and controls used in the study, there was no significant difference in age (p = 1.000). Regarding the marital status and the professional category, we did not found a significant difference in women with EP compared to those with IUP. Smoking at the time of conception was more found among in EP cases than controls (OR: 2.75, 95% CI: 1.37–5.49).

**Table 1:** Socio-demographic characteristics of enrolled participants.

Variables	Cases N = 88 n (%)	Controls N = 176 n (%)	OR (95%CI)	*P* value	
**Age (years)**					
**[18–23]**	10 (11.4)	21 (11.9)	Reference		1.000
**[23–28]**	24 (27.3)	47 (26.7)	0.93 (0.37–2.29)		
**[28–33]**	35 (39.8)	70 (39.8)	0.95 (0.40–2.24)		
**[33–38]**	13 (14.6)	26 (14.6)	0.95 (0.34–2.60)		
**[38–43]**	5 (5.7)	10 (5.7)	0.95 (0.25–3.53)		
**≥43**	1 (1.1)	2 (1.1)	0.95 (0.07–11.78)		
**Marital status**					
**Single**	26 (29.5)	45 (25.6)			0.556
**Married or living together**	62 (70.5)	131 (74.4)	0.81 (0.46–1.44)		
**Educational level**					
**Primary school**	14 (15.9)	11 (6.4)	Reference		<0.001
**Secondary school**	54 (61.4)	85 (48.3)	2.00 (0.84–4.73)		
**High school**	20 (22.7)	80 (45.5)	5.35 (2.10–13.64)		
**Professional category**					
**Professional and managerial occupations**	3 (3.4)	11 (6.3)	Reference		0.125
**Intermediates professions**	12 (13.6)	32 (18.2)	0.72 (0.17–3.06)		
**Services and sales staff**	38 (43.2)	51(29.0)	0.36 (0.09–1.40)		
**Unemployed**	35 (39.8)	82 (46.6)	0.63 (0.16–2.43)		
**Smoking**					
**Yes**	21 (23.9)	18 (10.2)	2.75 (1.37–5.49)		0.003
**No**	67 (76.1)	158 (89.8)			

OR odds ratio, CI Confidence interval.

### Reproductive factors

EP’s women were more likely to have had previous induced abortion than IUP controls (OR: 2.08, 95% CI: 1.21–3.57) (Table 2).

**Table 2:** Reproductive, gynaecological and surgical histories of enrolled participants.

Variables	Cases N = 88 n (%)	Controls N = 176 n (%)	OR (95%CI)	*P* value
**Reproductive history**				
**Previous miscarriage**				
**Yes**	32 (36.4)	51 (29.0)	1.40 (0.81–2.41)	0.223
**No**	50 (54.8)	129 (75.2)		
**Prior induced abortion**				
**Yes**	38 (43.2)	47 (26.7)	2.08 (1.21–3.57)	0.007
**No**	50 (56.8)	129 (73.3)		
**Parity**				
**0**	22 (25.0)	66 (37.5)	Reference	0.020
**1**	19 (21.6)	45 (25.6)	0.76 (0.36–1.59)	
**2–3**	35 (39.8)	51 (29.0)	0.38 (0.20–0.74)	
**4 or more**	12 (13.6)	14 (8.0)	0.34 (0.14–0.84)	
**Surgical history**				
**Previous tubal surgery**				
**Yes**	5 (5.7)	1 (0.6)	10.54 (1.21–91.67)	0.017
**No**	83 (94.3)	175 (99.4)		
**Gynaecological history**				
**Prior CT infection**				
**Yes**	50 (68.5)	44 (25.3)	6.42 (2.77–10.38)	<0.001
**No**	23 (31.5)	86 (48.9)		
**Prior PID**				
**Yes**	59 (67.0)	23 (13.1)	13.53 (7.24–25.26)	<0.001
**No**	29 (33.0)	153 (86.9)		
**Prior EP**				
**Yes**	6 (6.8)	2 (1,1)	6.36 (1.25–32.22)	0.018
**No**	82 (93.2)	174 (98.9)		
**Previous infertility**				
**Yes**	32 (36.4)	32 (18.2)	2.57 (1.44–4.58)	0.001
**No**	56 (63.6)	144 (81.8)		
**Documented tubal pathology**				
**Yes**	7 (38.9)	9 (13.8)	3.96 (1.21–12.89)	0.017
**No**	11 (61.1)	56 (86.2)		

OR odds ratio, CI confidence interval, CT *Chlamydia Trachomatis*, PID pelvic inflammatory disease, EP ectopic pregnancy.

### Surgical factors

Previous tubal surgery appeared to be more frequent in cases than controls (OR:10.54, 95% CI:1.21–91.67).

### Gynaecological factors

Prior CT infection (OR: 6.42, 95% CI: 2.77–10.38), prior PID (OR: 13.53, 95% CI: 7.24–25.26), previous infertility (OR: 2.57, 95% CI: 1.44–4.58) and documented tubal pathology (OR: 3.96, 95% CI: 1.21–12.89) were more likely among cases than controls (Table 2). Also, EP cases were more likely to have had prior EP compared to controls (OR: 6.36, 95% CI:1.25–32.22). (Table 2).

An age of sexual debut less than 16 years was more probable in EP women than in IUP ones (OR: 3.27, 95% CI: 1.70–6.28); as well as having had more than 5 lifetime sexual partners (OR: 4.00, 95% CI: 2.14–7.45). (Table 3).

**Table 3:** Previous sexual history, previous and current use of contraceptives.

Variables	Cases N = 88 n (%)	Controls N = 176 n (%)	OR (95%CI)	*P* value	
**Previous sexual history**					
**Age at sexual debut (years)**					
**<16**	26 (29.5)	20 (11.4)	3.27 (1.70–6.28)	<0.001	
**≥16**	62 (70.5)	156 (88.6)	0.30 (0.15–0.58)		
**Lifetime number of sexual partners**					
**>5**	32 (36.4)	22 (12.5)	4.00 (2.14–7.45)		<0.001
**≤5**	56 (63.6)	154 (87.5)	0.25 (0.13–0.46)		
Previous use of contraceptives*					
**IUD**					
**Yes**	2 (3.00)	4 (3.1)	0.96 (0.17–5.43)		1.000
**No**	65 (97.0)	126 (96.9)			
**LNG-EC**					
**Yes**	22 (32.8)	31 (23.8)	1.56 (0.81–2.99)		0.178
**No**	45 (67.2)	99 (76.2)			
****COCs					
**Yes**	3 (4.5)	5 (3.8)	1.17 (0.22–5.06)		1.000
**No**	64 (95.5)	125 (96.2)			
**POPs**					
**Yes**	6 (9.0)	5 (3.8)	2.45 (0.72–8.37)		0.189
**No**	61 (91.0)	125 (96.2)			
**DMPA**					
**Yes**	13 (19.4)	10 (7.7)	2.88 (1.19–6.99)		0.015
**No**	54 (80.6)	120 (92.3)			
**Progestin-only implants**					
**Yes**	2 (3.0)	8 (6.2)	0.46 (0.09–2.27)		0.499
**No**	65 (97.0)	122 (93.8)			
Current use of contraceptives*					
**LNG-EC**					
**Yes**	10 (14.9)	3 (2.3)	7.42 (1.96–28.01)		0.001
**No**	57 (85.1)	127 (97.7)			

OR odds ratio, CI confidence interval, IUD intra-uterine device, LNG-EC levonorgestrel-only pills for emergency contraception, COCs combined oral contraceptives, POPs progestin-only pills, DMPA depot medroxyprogesterone acetate

*Women who did not use contraceptives are not included here (n = 21 cases and n = 46 controls).

### Contraceptive factors

Previous use of progestin-only injectable DMPA was more likely in EP cases, than controls (OR: 2.88, 95% CI:1.19–6.99) (Table 3).

By contrast, current use of LNG-EC was significantly more probable among EP cases than controls (OR: 7.42, 95% CI: 1.96–28.01). We did not find any women who were taking progestin-only injectable or implant, COCs or POPs during the cycle of conception.

The results of the multivariable logistic regression analysis are shown in Table 4. The model included smoking at the time of conception, prior PID, prior EP, prior induced abortion, prior tubal surgery, age at sexual debut, previous use of DMPA and current use of LNG-EC. Factors found to be independently associated to EP were: prior PID (AOR: 13.03; 95% CI 6.19–27.42), current use of LNG-EC (AOR: 10.15; 95% CI 2.21–46.56), previous use of DMPA (AOR: 3.01; 95% CI 1.04–8.69) and smoking at the time of conception (AOR: 2.68; 95% CI 1.12–6.40).

**Table 4:** Multivariable logistic regression analysis for independent risk factors of EP.

Variables*	AOR (95% Ci)	P value
**Smoking at the time of conception**		
Yes	2.68 (1.12–6.40)	0.026
No		
**Prior PID**		
Yes	13.03 (6.19–27.24)	<0.001
No		
**Prior EP**		
Yes	1.88 (0.21–16.80)	0.568
No		
**Prior induced abortion**		
Yes	1.79 (0.89–3.60)	0.101
No		
**Prior tubal surgery**		
Yes	0.99 (0.06–14.70)	0.997
No		
**Age at sexual debut (years)**		
<16	1.70 (0.73–3.94)	0.212
≥16		
**Previous use of DMPA**		
Yes	3.01 (1.04–8.69)	0.041
No		
**Current use of LNG-EC**		
Yes	10.15 (2.21–46.56)	0.003
No		

AOR adjusted odds ratio, CI confidence interval, EP ectopic pregnancy, PID pelvic inflammatory disease, DMPA depot medroxyprogesterone acetate, LNG-EC, levonorgestrel-only pills for emergency contraception

*All included variables had were statistically significant at *P* <0.05

## Discussion

This study found a strong independent association between prior PID and EP occurrence (Table 4). Our finding aligns with numerous studies which identified PID as a major risk factor for EP [19,25,26], because of scarring, resulting in tubal obstruction that interfere**s** with egg’s capture and migration, but also with spermatozoa migration [25]. Such finding could reflect an important rate of late diagnosis and untreated acute pelvic infections, in our country, probably due to poverty.

Consistent with previous studies [27–29], our study identified an independent significant connection between EP and the use of LNG-EC during the cycle of conception. It has been suggested that higher levels of progesterone could alter ciliary beat function and smooth muscles contractility of Fallopian Tubes (FTs), and the high serum peak of LNG observed after an administration of a single dose of LNG-EC could possibly result in a tubal motility decline; thus, increasing the risk of EP [29–31]. Thereby, if LNG-EC is taken at a time when it is ineffective in preventing pregnancy, the plasma concentration of LNG-EC might still remain high during the time of embryo-tubal transport due to its half-life of 24 h; therefore, the chance of embryo-tubal implantation increases with declined tubal motility [21].

Previous use of DMPA was also found to have an independent relationship with EP occurrence. It has been shown that during the menstrual cycle, level of Estradiol Receptors (ERs) -mostly localized on FT’s ciliated, secretory epithelial and smooth muscles cells- fluctuate in response of high circulating Estradiol (E2) levels [32–34]. Adrenomedullin (ADM), a polypeptide structurally similar to calcitonin, expressed in epithelial cells of the human and rat FTs increase and decrease along with circulating E2 levels during the menstrual cycle and increases ciliary beat frequency [35–36]. With these results, we suggest that by its antiestrogen activity (including inhibition of ERs, decreasing levels of circulating E2), DMPA could decrease ER’s expression in FT cells, resulting in a loss of ADM expression and subsequent dysfunction in ciliary beat and tubal motility.

Such finding may also be due to a lack of barrier contraceptive use, such as condoms because, using a simple and safe contraceptive, women feel well protected from pregnancies. Yet with unprotected sex, there is a risk of STIs and subsequent PID.

Another independent factor found to be associated with EP in our study was smoking at the time of the conception. This has been demonstrated in other countries [37,38]; but to our knowledge, none in Cameroon. Shaw et al. [38] reported that cotinine (the most abundant metabolite of nicotine) increased the expression of prokineticin receptor-1 (PROKR1), a regulator of smooth muscle contractility and genes that are important in implantation, in the FT. In this sense, they hypothesized that smoking predisposes women to EP by altering tubal PROKR1 expression resulting in changes in FT function. Therefore, awareness of women on smoking dangers and encouraging them on smoking avoidance and cessation is necessary.

Other variables were significantly associated with EP occurrence, but not independently after the multivariable logistic regression analysis; probably due to confounding variable effect, such as STDs. STDs could have been the confounder increasing the relationship between these factors and EP occurrence. However, it would be important to pay attention to these variables in order to enhance prevention in our resource-limited country.

In our findings, prior CT infection, previous infertility, documented tubal pathology and prior EP were found to increase EP risk. These results are consistent with those of previous studies, which indicated that EP risk was higher in women with prior CT infection [19,25,26], as well as previous infertility, documented tubal pathology [17,20] and prior EP [20,39]. The healing process that follows *C*. *Trachomatis* infection can result in alterations of tubal mucosa and limit tubal motility [19]. This could lead to PID and subsequently, interfere with the egg’s capture or with spermatozoa’s migration [19]. Infertility may result from these tubal damages. The association between prior EP and the new EP may reflect persistent exposure to pre-existent risk factors, especially those that cause tubal dysfunction.

The study also revealed a significant connection between prior induced abortion and EP occurrence. This finding seem**s** to be different from those in countries where abortions are legalized [40,41]. In Cameroon, as in most African countries where abortions are not legalized, most abortions are illegal and usually occur in poor aseptic conditions [42]. Thus, increasing post-abortion sepsis risk and subsequent PID. There are others African publications reporting a relationship between prior induced abortions and EP [25,42]. Clearly, this is an area deserving further attention because such finding may reflect potential inadequacies of the national family planning policy in our country. This thus evokes an increased need for a popularization of modern contraceptives use among Cameroonian women, in order to prevent unwanted pregnancies, which motivate the decision to have an abortion. On the other hand, it suggests that there should be more training medical staff on post-abortion care in primary and secondary hospitals, to improve the management of abortions related complications.

We found a significant relationship between previous tubal surgery and EP. Our results are in line with those of a meta-analysis including 27 case-control studies and 9 cohort studies, which reported previous tubal surgery to be strongly associated with the occurrence of EP [17].

Have had first sexual intercourse at less than 16 years was significantly associated with EP occurrence, and having more than 5 lifetime’s sexual partners. The early age at sexual debut has long been identified as a determinant for STDs, notably bacterial infections for both behavioral and biological factors [43]. In line with this, Cates asserted that the earlier sexual activity is started, the longer the time of exposure to different sexual partners because, for adolescents, adolescence represents a time for sexual experimentation [44]. Concerning this, Aral has suggested that the risk of STDs in women having cumulated several sexual partners was associated to the fact that they tend to have large number of current partners [45]. In Cameroon, Nkwabong et al. in their cross sectional descriptive study including 70 patients diagnosed with acute PID of 1344 women who were seen for gynaecologic problems, 45 (64.3%) had many sexual partners; the reason, being probably the fact that they depend on their partners due to underemployment and poverty in Cameroon [46]. We believe that reducing the costs in genital infections examinations for adolescents at least in health services, might help improve the early diagnosis and treatment of STDs in order to reduce subsequent sequelae such as PID, infertility, and EP.

No significant association was found between previous use of LNG-EC, COCs, POPs and EP. Probably the number of the studied participants was too small for any meaningful deductions. In fact, in Cameroon, modern contraceptives are used by a minority of population. According to the Demographic and Health survey and multiple indicators realized in Cameroon [47], among non-pregnant women aged between 15–49 years, 16% were using at least one contraceptive method. The injectable use rate was 2%, that of pills 2% and for other modern contraceptives, less than 1%. This could be linked to prejudices, socioeconomic and cultural barriers, lack of information and ignorance of some Cameroonians, which are abord key obstacles to adoption of contraception. This suggest**s** a need for increasing access to and changing social norms about modern contraception.

## Conclusions

The major risk factor identified in this study is prior PID. In order to control these infections, a strengthening of health education in Cameroonian women of childbearing age, by encouraging them to reduce risky sexual behaviours (such as having multiple sex partners or unprotected sex without regular screening for STIs) is essential. Furthermore, policies on reducing the cost of screening and treatment of STIs should be put in place, especially for adolescents to promote timely access to healthcare for reducing chances of PID and subsequent EP.

Besides, findings of current use of LNG-EC, previous use of DMPA and smoking at the time of conception as risk factors, are newly identified in our context. All this highlights that physicians should pay attention to women’s counselling about EP (in the face of this multiplicity of risk factors reflecting increased susceptibility to EPs nowadays in our country). In addition, an increased need for easier access to equipment and skills for earlier diagnosis of EP is emerging, in a view of managing EPs, with less implication on fertility of Cameroonian women.

### Strengths and limitations

The study was carried out across the two main referral hospitals for gynecologic and obstetric emergencies in Yaoundé; well representing the urban and rural population. The cases and controls were from the same source of population, which makes this comparison valid and the findings are generalizable to all EP in our context. However, as a hospital-based case-control study we acknowledge for recall and selection bias.

## Supporting information

S1 Table Dataset. (XLS)
